# A Novel Three-Stage Framework for Association Analysis Between SNPs and Brain Regions

**DOI:** 10.3389/fgene.2020.572350

**Published:** 2020-09-24

**Authors:** Juan Zhou, Yangping Qiu, Shuo Chen, Liyue Liu, Huifa Liao, Hongli Chen, Shanguo Lv, Xiong Li

**Affiliations:** School of Software, East China Jiaotong University, Nanchang, China

**Keywords:** imaging genetics, data mining, single nucleotide polymorphism, association analysis, Alzheimer's disease

## Abstract

**Motivation:** At present, a number of correlation analysis methods between SNPs and ROIs have been devised to explore the pathogenic mechanism of Alzheimer's disease. However, some of the deficiencies inherent in these methods, including lack of statistical efficacy and biological meaning. This study aims at addressing issues: insufficient correlation by previous methods (relative high regression error) and the lack of biological meaning in association analysis.

**Results:** In this paper, a novel three-stage SNPs and ROIs correlation analysis framework is proposed. Firstly, clustering algorithm is applied to remove the potential linkage unbalanced structure of two SNPs. Then, the group sparse model is used to introduce prior information such as gene structure and linkage unbalanced structure to select feature SNPs. After the above steps, each SNP has a weight vector corresponding to each ROI, and the importance of SNPs can be judged according to the weights in the feature vector, and then the feature SNPs can be selected. Finally, for the selected feature SNPS, a support vector machine regression model is used to implement the prediction of the ROIs phenotype values. The experimental results under multiple performance measures show that the proposed method has better accuracy than other methods.

## Introduction

Alzheimer's disease (AD) is characterized by complex pathogenesis, slow progression of disease, irreversible pathologic nature, and no obvious organic lesions in the early stage. Mild cognitive impairment (MCI) is considered an early stage of AD. Without scientific intervention and treatment, early patients with AD or MCI will continue to deteriorate, seriously affecting their quality of life. Therefore, timely detection of early AD and early scientific intervention are of great significance for the prevention and treatment of AD. Single nucleotide polymorphism (SNPs) is a polymorphism at the DNA level, which is the key source of the occurrence and development of AD. Magnetic Resonance Imaging (MRI) technology has been proved to be an effective method for the detection of a variety of mental diseases such as AD. The candidate brain regions that may be related to AD are called region of interest (ROIs) by researchers, and according to the morphological characteristics such as density and volume of ROIs, the brain structure or function of individuals is judged to be abnormal (Alfaro-Almagro et al., 2018). At present, some methods of correlation analysis between SNPs and brain ROIs have been widely used to explore the pathogenesis and risk assessment of Alzheimer's disease. For example, association analysis was carried out between the candidate gene *APOE* and target phenotypes such as medial temporal lobe atrophy, hippocampal volume, and hippocampal shape change trajectory (Andrawis et al., 2012; Jack et al., 2012), and the results showed a significant negative correlation (Tosun et al., 2010). Yan et al. introduced the sparse canonical correlation analysis model into the algorithm of priori information induction, and then applied the algorithm to carry out a correlation analysis between APOE gene and several candidate target phenotypes (Yan et al., 2014). Hao et al. proposed a tree-structured sparse learning model for the correlation analysis of candidate genetic variation and MRI brain imaging regions (Hao et al., 2018). This strategy partially ignores the interrelationships between brain regions and may miss other important genetic variations that have not yet been reported.

In recent years, genome wide association study (GWAS) has been applied to the study of different complex diseases globally (Hu et al., 2018; Zhou et al., 2018), and the relevant susceptible SNPs have been accurately identified and included in the GWAS Catalog (Welter et al., 2014). With the generation of high-throughput whole-genome sequencing data, the role of data-driven genome-wide association research method on the pathogenesis of AD becomes more and more obvious. For example, GWAS was used to identify the susceptibility genes significantly related to AD (Ewers et al., 2006), such as AIV, APP, A2M and APOEε 4, which are involved in the regulation of important biological processes such as Aβ degradation, lipid metabolism and APP metabolism, respectively. However, with further research, it was found that the experimental results obtained by traditional GWAS were difficult to repeat, with low explanatory power and lack of heritability. For example, some common or rare variants associated with AD have been identified (Saykin et al., 2015; Marei et al., 2016), in which the APOEε 4 allele has been shown to be significantly associated with AD in most studies, but only 50% of AD patients carry the APOEε 4 allele. The study (Karch et al., 2014) indicates that there are other genetic factors involved in the occurrence and development of AD. According to recent studies (Ridge et al., 2013), about 33% of the variation of the AD phenotype can be explained by common variation sites, among which APOE accounts for 6%, while other known variation sites account for 2%, which means that about 25% of the variation of the AD phenotype is still not explained by common variation sites. Currently, there are also genome-wide association studies with candidate phenotypes. For example, Li et al. carried out genome-wide association studies on quantitative features of AV-45 and found 8 pairs of epistatic effects (Li et al., 2015). Saykin et al. conducted a genome-wide association study with hippocampal volume and hippocampal gray matter density as the target phenotype, identifying genes such as CDH8, MAD2L2, QPCT, and GRB2 that had not been reported in previous studies (Saykin et al., 2015). Due to the large number of SNPs in the whole genome, epistasis between SNPs is often ignored in some studies, resulting in insufficient correlation efficiency. Stein et al. investigated the association between about 448,000 SNPs in the genome-wide range and 31,000 brain voxels in 740 ADNI volunteers (Stein et al., 2010). However, the sample size of this study is small, facing the challenge of small sample with high dimension and insufficient statistical significance.

A regression model based on L_1_ paradigm penalty constraints (Kohannim et al., 2012; Yang et al., 2015) has been successfully applied to multivariate genetic data analysis. However, these methods ignore important underlying interacting relationships between the SNPs. Based on this, Silver et al. proposed a group sparse (Yuan and Lin, 2006; Silver et al., 2012, 2013) model to solve the image genetics problem. Further, Wang et al. proposed a group sparse multi-task regression model G-SMuRFS to extract feature SNP (Wang et al., 2012), which makes full use of the group structure and is conducive to improving regression performance. G-SMuRFS only furnishes a point estimate of the regression coefficients; techniques for conducting statistical inference are not provided. Greenlaw et al. proposed a Bayesian group sparse multi-task regression model for imaging genetics to overcome the limitation (Greenlaw et al., 2017).

In view of the shortcomings of current image genetics methods, this paper proposes a new solution approach from the perspectives of prior information fusion and feature fusion and applies it to SNPs-ROIs correlation analysis. In this study, a three-stage framework for correlation analysis of SNPs and brain regions was proposed. The framework includes: in the filtering stage, hierarchical clustering algorithm was adopted to identify the linkage unbalanced structure between two sites, and to preliminarily eliminate the redundant and noisy SNPs; In order to fuse the prior information such as the distribution of SNPs on genes and the linkage imbalance among SNPs, the group sparse model was adopted in the feature selection stage to contain the grouping characteristics of feature SNPs. Finally, the support vector regression model was used to improve the regression performance of SNP and ROI. Experimental results show that the error of this method is lower than that of other regression analysis methods.

## Experimental Data and Evaluation Data

### Data Source-ADNI Database

ADNI (Alzheimer's Disease Neuroimaging Initiative) database (http://adni.loni.usc.edu/) is one of the most widely used and reliable international sources of data for AD and MCI experiments. ADNI integrates genetic, imaging, and clinical data into a data platform for analysis, so as to facilitate global researchers to further study the occurrence and development mechanism of AD. In this paper, we use the dataset obtained from ADNI database, which includes both genetic and structure magnetic resonance imaging (MRI) data and is similar to a dataset analyzed by Wang et al. (2012). Our goal is to select feature SNPs with a high degree of association with ROIs by association analysis between SNPs and ROIs. Then utilizing regression models to analyze the degree of association between feature SNPs and ROIs. For more information about ADNI, please visit www.adni.loni.usc.edu.

### Problem Description

(1) $$\begin{array}{l} \left[\underset{Xn*p}{\underset{︸}{\begin{array}{cccc} s11 & \cdots & \cdots & s1p\\ s21 & & & \vdots \\ \vdots & & & \vdots \\ sn1 & \cdots & \cdots & snp \end{array}}}\;\underset{Yn*q}{\underset{︸}{\begin{array}{ccc} \;r11 & \cdots & r1q\\ \vdots & & \vdots \\ \vdots & & \vdots \\ rn1 & \cdots & rnq \end{array}}}\right]\\ \;\begin{array}{l} Min\;|Y-f(S)|\\ s.t\;Min|S| \end{array} \end{array}$$

where *X*_n*p_ matrix represents the alleles on candidate SNPs, and *n* represents the number of samples, *p* represents the number of SNPs. *S*_*i, j*_ ∈ {0, 1, 2}, ‘0’ denotes the wild homozygous type, ‘1’ represents the heterozygous type, and ‘2’ represents the mutant homozygous type. *Y*_n*q_ represents ROIs matrix, where *n* represents the number of samples, *q* represents the number of ROIs, and its value is a continuous real number. *f* (*S*) represents a prediction model, where *S* represents the set *S*. Formula (1) is the mathematical model of this problem, and the task is to find a minimum SNPs set *S* to make its predicted value of ROIs as close as possible to the real ROIs value. The dataset used in this paper contains 632 samples, each of which has 486 SNPs and 56 ROIs.

### The Evaluation Measures

In order to objectively and comprehensively evaluate the ROIs prediction performance of the method in this paper, this section adopts multiple groups to evaluate, such as Formula (2) – (7), which including Mean Absolute Error (MAE), Root Mean Square Error (RMSE), Median Absolute Error (MeAE), Mean Absolute Percentage Error (MAPE), *R*^2^ and Root Mean Square Percentage Error (RMSPE).

(2) $$\begin{array}{r} MAE=\frac{1}{\text{n}}\overset{n}{\underset{i\;=\;1}{\sum }}|f_{i}-y_{i}| \end{array}$$

(3) $$\begin{array}{r} RMSE=\sqrt{\frac{1}{\text{n}}\overset{n}{\underset{i\;=1}{\sum }}{\left(f_{i}-y_{i}\right)}^{2}} \end{array}$$

(4) $$\begin{array}{r} MeAE=\text{median}\left(f_{i}-y_{i}\right) \end{array}$$

(5) $$\begin{array}{r} MAPE=\frac{100}{n}\overset{n}{\underset{i\;=1}{\sum }}|\frac{y_{i}-f_{i}}{y_{i}}| \end{array}$$

(6) $$\begin{array}{r} R^{2}=1\text{-}\frac{\overset{n}{\underset{\text{i }=\;1}{\sum }}{\left(y_{i}-f_{i}\right)}^{2}}{\overset{n}{\underset{i\;=1}{\sum }}{\left(y_{i}-\bar{y}\right)}^{2}} \end{array}$$

(7) $$\begin{array}{r} RMSPE={\sqrt{\frac{1}{\text{n}}{\overset{n}{\underset{i\;=1}{\sum }}\left(\frac{y_{i}-f_{i}}{y_{i}}\right)}^{2}}}^{} \end{array}$$

where *f* represents the predicted value and *y* represents the actual value.

## Methods

**Figure 1:**
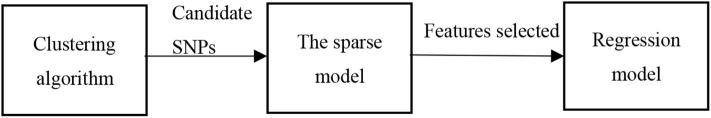
A three-stage frame diagram.

The study of the association between millions of SNPs in the whole genome and ROIs in the brain region is conducive to the discovery of pathogenic genes, but the large number of SNPs and higher order interactions between them lead to combinational explosion. This paper proposes a new SNPs-ROIs correlation analysis framework shown in Figure 1, which is divided into three modules. Firstly, clustering algorithm is used to identify potential linkage unbalanced structures to preliminary filter the redundant noise between two sites; Then the group sparse model was used to extract the feature sites. Finally, a regression model was constructed to predict the phenotypic values of the brain regions of the samples.

### Hierarchical Clustering Algorithm

High-throughput sequencing technology has produced large-scale omics data, and clustering algorithm is a common mining algorithm, such as tumor subtype recognition, gene co-expression module analysis, and so on (Qiu, 2020). Hierarchical clustering is one of the most widely used classical Clustering methods. This method usually uses tree structure to describe the class membership relationship among members, so its clustering process and clustering results can be visualized through the form of a tree. Hierarchical clustering algorithm can be divided into two specific processes: bottom-up aggregation and top-down splitting. The initial state of clustering in the aggregation hierarchy is that a single sample forms a cluster separately, and then the two clusters with the smallest distance are merged by calculating the distances between all the class clusters, and then the two clusters with the smallest distance are iteratively searched for and merged until the exit condition is satisfied. The following distance methods are commonly used for clustering: the shortest distance method, maximum distance method, centroid distance method, unweighted average distance, least variance method, center of gravity method and so on. In this study, we compare the effectiveness of the five previous distance measurement methods in hierarchical clustering and ultimately find which method is the most applicable. In each step of clustering, the same combination is carried out until the expected number of classes. On the contrary, split hierarchical clustering first initializes all objects into a class cluster, and then divides the class cluster according to the partition rule until the exit condition is satisfied. The exit condition can be either a distance threshold or the number of class clusters to be satisfied.

### Feature SNP Selection Based on Group Sparse Model

Association studies based on univariate usually assume that SNPs are independent from each other, so they are statistically analyzed separately with the target phenotype. But with millions of SNPs distributed throughout the genome, association analysis alone with the target phenotype can lead to problems such as multiple testing problem. The feature SNP selection process can select a feature SNP subset from millions of SNPs, which can not only effectively express the phenotypic information of brain ROIs, but also make the feature subset as compact as possible, that is, contain as little redundant information as possible. The common embedded feature selection method is to use cost function and regularization to achieve this goal. Theoretically, the L_0_ norm can be used to describe the selected number of features intuitively, but it is usually difficult to optimize the L_0_ norm. Therefore, L_1_ norm and L_2_ norm are commonly used in the actual modeling process. LASSO (Least absolute shrinkage and selection operator) is a typical application of L_1_ norm. It achieves sparse expression and feature selection by introducing L_1_ regularization to construct a minimization target function model. Ridge regression (RR) introduces L_2_ norm into the objective function.

Phenotypic data of brain ROIs are continuous values, and the most intuitive way is to describe the relationship between SNPs and ROIs by linear regression (LR). However, RR can not only avoid overfitting, but also promote numerical stability (de Vlaming and Groenen, 2015; Greenlaw et al., 2017). Given the ADNI participant $\left\{x_{1},\cdots \;,x_{n}\right\}\subseteq {ℜ}^{d}$ and the selected imaging phenotype $\left\{y_{1},\cdots \;,y_{n}\right\}\subseteq {ℜ}^{c}$, *n* is the number of samples, *d* is the number of SNPs (feature dimension), and *c* is the number of ROIs (brain region phenotype). Then, the ridge regression model is shown in equation (8).

(8) $$\begin{array}{r} \underset{w}{\min}{\overset{n}{\underset{i\;=\;1}{\sum }}\|W^{T}x_{i}-y_{i}\|}^{2}+\gamma {\overset{d}{\underset{i\;=\;1}{\sum }}\|w^{i}\|}^{2} \end{array}$$

Where *W* represents the weight matrix of the i-th SNP in predicting ROIs in the j-th brain region, and γ is the weight parameter. The larger the parameter is, the more important the role of the regularization term in the objective function is, that is, to improve the sparsity of the weighted model, and on the contrary, the more emphasis is placed on the degree of fitting between the predicted value and the actual value.

However, the ridge regression model has some shortcomings in the actual analysis of the correlation between SNPs and ROIs (Du et al., 2019, April): first, the weight matrix *W* is not sparse, and all SNPs participate in the prediction of brain ROIs phenotype through the weight matrix, although the weight of some SNPs is very sparse. However, only a small number of SNPs are actually related to target ROIs. Secondly, similar to the linear regression model, the tasks of the ridge regression model are decoupled, that is, each task can be learned separately. Finally, the model ignores the group structure between SNPs. In fact, there is not only a linkage disequilibrium (LD) between SNPs (Slatkin, 2008), but also an interaction between the genes in which SNPs are located. LD refers to the non-random association between different sites, and the distribution of sites with high LD is more stable in the population. Therefore, it is necessary to consider the grouping structure of SNPs. In addition, the human brain is composed of multiple brain regions, which cooperate with each other to accomplish complex functions. For example, the function of episodic memory requires the combination of the medial temporal lobe (MTL) structure and brain regions such as the medial and lateral parietal lobes and the prefrontal cortex. Therefore, the accurate prediction of subjects' brain function often involves the combination of multiple brain regions and their related biomarkers.

Therefore, for the above reasons, the group structure of SNP can not only transmit important biological information, but also help to improve the statistical efficiency. Each SNP can be regarded as a genetic characteristic and each ROI phenotype as a response variable, so the regression relationship between multiple SNPs and an ROI is a learning task, and the study on the correlation between multiple SNPs and multiple ROIs is called multi-task regression. Wang et al. proposed a group sparse multi-task regression model G-SMuRFS to extract feature SNP (Wang et al., 2012). Researchers believe that SNPs that are physically close to each other on the same chromosome are often inherited and related. Making full use of the group structure is conducive to improving the regression performance and according to the biological significance. Therefore, G-SMuRFS first divided SNPs into *k* groups according to division rules $\prod ={\left\{\pi_{k}\right\}}_{k\;=\;1}^{K}$, among them ${\left\{W^{i}\right\}}_{i\;=\;1}^{m_{k}}\in \pi_{k}$, *m*_*k*_ is the number of SNPs in π_*k*_. Intuitively, there are two simple grouping rules. One is to set a distance threshold and divide SNPs less than the threshold into the nearest or subordinate genes. The other is to use the LD criterion *r*^2^. The larger the value is, the higher the linkage between the two SNPS is, and the more likely they are to be in the same group. In this paper, the sites of *r*^2^ ≥ 0.2 were grouped.

For the sake of description, write the matrix in bold uppercase and the vector in bold lowercase. Given a matrix *M* = *m*_*ij*_, the *i*-th row and *j*-th column are denoted as *m*^*i*^ and *m*_*j*_, the matrices of Frobenius norm an ℓ_2, 1_ norm are defined as ${\left\|M\right\|}_{2,1}=\sum_{i}\sqrt{\sum_{j}m_{ij}^{2}}=\sum_{i}{\left\|m^{i}\right\|}_{2}$ and ${\left\|M\right\|}_{F}=\sqrt{\sum_{i}\|m^{i}{\|}_{2}^{2}}$. Therefore, the group sparse model is expressed as equation (9).

(9) $$\underset{W}{\min}\sum_{i\;=\;1}^{n}{\left\|W^{\text{T}}x_{i}-y_{i}\right\|}_{2}^{2}+\gamma \sum_{k\;=\;1}^{K}\sqrt{\sum_{i\in \pi_{k}}\sum_{j\;=\;1}^{c}w_{ij}^{2}}$$

In the equation: $W=\left[\begin{array}{c} W^{1}\\ \cdots \\ W^{K} \end{array}\right],W^{k}\in {ℜ}^{m_{k}\times c}\left(1\leq k\leq K\right)$

The matrix norm can be used to rewrite equation (9) into equation (10).

(10) $$\begin{array}{r} \underset{W}{\min}\overset{n}{\underset{i\;=\;1}{\sum }}{\left\|W^{T}x_{i}-y_{i}\right\|}_{2}^{2}+\gamma {\overset{K}{\underset{k\;=\;1}{\sum }}\|W^{k}\|}_{F} \end{array}$$

Because of:

$\begin{array}{l} X=\left[x_{1},\cdots x_{n}\right],Y=\left[y_{1},\cdots y_{n}\right],\\ {\left\|W\right\|}_{G_{2.1}}=\sum_{k=1}^{K}\sqrt{\sum_{i\in \pi_{k}}\sum_{j\;=\;1}^{c}W_{ij}^{2}}=\sum_{k\;=\;1}^{K}{\left\|W^{k}\right\|}_{F} \end{array}$ we can get equation (11):

(11) $$\begin{array}{r} \underset{W}{\min}\|W^{T}X-Y{\|}_{F}^{2}+\gamma \|W{\|}_{G_{2,1}} \end{array}$$

Although Formula (11) considers the inter-group structure of SNP data through the proposed *G*_2, 1_ norm, the feature selection between tasks has not been completely solved. In an important group structure, some features may be irrelevant. On the other hand, in less important groups, some features may be important. Therefore, the Formula (11) model is implemented with additional structural sparsity, and the characteristics of multiple tasks are selected jointly through the regularization of ℓ_2, 1_ norm:

(12) $$\begin{array}{r} \underset{W}{\min}\gamma \overset{n}{\underset{i\;=\;1}{\sum }}{\left\|W^{T}x_{i}-y_{i}\right\|}_{2}^{2}+\gamma_{1}\overset{K}{\underset{k\;=\;1}{\sum }}{\left\|W^{k}\right\|}_{F}+\gamma_{2}\overset{d}{\underset{i\;=\;1}{\sum }}{\left\|w^{i}\right\|}_{2} \end{array}$$

The matrix form can be simply rewritten as:

(13) $$\underset{W}{\min}\sum_{i\;=\;1}^{n}{\left\|W^{T}X-Y\right\|}_{F}^{2}+\gamma_{1}{\left\|W\right\|}_{G_{2,1}}+\gamma_{2}{\left\|W\right\|}_{2,1}$$

In Formula (13), the first item is to measure the structural error between the return value and the real value when measuring the SNP regression ROI phenotype. In the second item, a set of features in the task was first identified and then all their regression coefficients were coupled together. Because of the genetic linkage, the item incorporated SNP grouping information. The third item penalizes all regression coefficients of a single feature to select features that span multiple learning tasks. Existing algorithms usually need to reformulate sparsity problems such as the Second-Order Cone Programming (SOCP) or Semi-definite Programming (SDP), which can be solved by internal point method or beam method (Wang et al., 2012). However, solving SOCP or SDP is computationally expensive, which limits their use in practice. Therefore, since the number of genetic markers can be very large, an efficient algorithm such as Formula (13) is required. Wang et al. proposed an efficient algorithm to solve the objective function in Formula (13). In this paper, the G-SMURFS method is used as the feature extraction process in the three-stage analysis framework. In order to ensure the narrative integrity, the derivation process is described in detail below.

Take the derivative of *W*, set the derivative to 0, and you get equation (14).

(14) $$\begin{array}{r} XX^{T}W-XY^{T}+\gamma_{1}DW+\gamma_{2}\tilde{D}W=0 \end{array}$$

Where *D* is a block diagonal matrix, and the *k*-th block is $\frac{1}{2{\left\|W^{k}\right\|}_{F}}$, *I*_*k*_ is the identity matrix of size *m*, $\tilde{D}$ is a diagonal matrix, and its i-th diagonal element is $\frac{1}{2{\left\|W^{2}\right\|}_{2}}$, we can do the following derivation:

Suppose a matrix:

$$\begin{array}{l} A=\left(\begin{array}{ccc} a_{11} & \ldots & a_{1n}\\ \vdots & \ddots & \vdots \\ a_{m1} & \cdots & a_{mn} \end{array}\right),X=\left[\begin{array}{c} x_{1}\\ \vdots \\ x_{n} \end{array}\right]b=\left[\begin{array}{c} b1\\ \vdots \\ b_{n} \end{array}\right], \end{array}$$

Then:

$A\;\cdot \text{X}-b=\left[\begin{array}{l} a_{11}x_{1}+a_{12}x_{2}+\cdots +a_{1n}x_{n}-b_{1}\\ a_{12}x_{1}+a_{22}x_{2}+\cdots +a_{2n}x_{n}-b_{2}\\ \cdots \cdots \cdots \cdots \cdots \cdots \cdots \cdots \cdots \cdots \cdots \cdots \cdots \cdots \\ a_{m1}x_{1}+a_{m2}x_{2}+\cdots +a_{mn}x_{n}-b_{n} \end{array}\right]$

According to the paradigm definition,

$$\begin{array}{l} \;{\left\|X\right\|}_{2}=\sqrt{{x_{1}}^{2}+{x_{2}}^{2}+\cdots +{x_{n}}^{2}}\\ {{\left\|X\right\|}_{2}}^{2}={x_{1}}^{2}+{x_{2}}^{2}+\cdots +{x_{n}}^{2} \end{array}$$

Then$\|AX-b{\|}_{2}^{2}={\left(a_{11}x_{1}+a_{12}x_{2}+\cdots +a_{1n}x_{n}-b_{1}\right)}^{2}+\cdots +{\left(a_{m1}x_{1}+a_{m2}x_{2}+\cdots +a_{mn}x_{n}-b_{n}\;\right)}^{2}$

Take the partial derivative of the matrix *X*:

$$\begin{array}{l} \nabla_{x}\|AX-b{\|}_{2}^{2}=\left[\begin{array}{c} \nabla_{x_{1}}\|AX-b{\|}_{2}^{2}\\ \nabla_{x_{2}}\|AX-b{\|}_{2}^{2}\\ \vdots \\ \nabla_{x_{2}}\|AX-b{\|}_{2}^{2} \end{array}\right] \end{array}$$

Among them:

$$\begin{array}{l} \begin{array}{c} \nabla_{x_{1}}\|AX-b{\|}_{2}^{2}=2\left(a_{11}x_{1}+a_{12}x_{2}+\cdots +a_{1n}x_{n}-b\right)a_{11}+\\ 2\left(a_{21}x_{1}+a_{22}x_{2}+\cdots +a_{2n}x_{n}-b\right)a_{21}+\cdots \\ +2\left(a_{m1}x_{1}+a_{m2}x_{2}+\cdots +a_{mn}x_{n}-b\right)a_{m1} \end{array} \end{array}$$

Formulas such as $\nabla_{x_{n}}\|AX-b{\|}_{2}^{2}$ can also be analogized in turn. It can be found that:

$$\begin{array}{l} \begin{array}{c} \nabla_{x}\|AX-b{\|}_{2}^{2}=2\left[\begin{array}{c} a_{11}a_{21}\cdots a_{m1}\\ a_{12}a_{22}\cdots a_{m2}\\ \cdots \cdots \cdots \cdots \cdots \cdots \cdots \\ a_{1n}a_{2n}\cdots a_{mn} \end{array}\right]\cdot \end{array}\\ \begin{array}{c} \left[\begin{array}{c} a_{11}x_{1}+a_{12}x_{2}+\cdots +a_{1n}x_{n}-b\\ a_{21}x_{1}+a_{22}x_{2}+\cdots +a_{2n}x_{n}-b_{2}\\ \cdots \cdots \cdots \cdots \cdots \cdots \cdots \cdots \cdots \cdots \cdots \cdots \cdots \cdots \cdots \cdots \cdots \\ a_{m1}x_{1}+a_{m2}x_{2}+\cdots +a_{mn}x_{n}-b_{n} \end{array}\right]\\ =2A^{T}\left(AX-b\right) \end{array} \end{array}$$

According to the property: matrix transpose does not change the value of the norm, ${{\left\|A-B\right\|}_{2}}^{2}={{\left\|{\left(A-B\right)}^{T}\right\|}_{2}}^{2}$

Then ${{\left\|W^{T}X-Y\right\|}_{2}}^{2}={{\left\|X^{T}W-Y^{T}\right\|}_{2}}^{2}$, we can know from Formula (15):

(15) $$\begin{array}{l} \begin{array}{c} {{\left\|W^{T}X-Y\right\|}_{2}}^{2}={{\left\|X^{T}W-Y^{T}\right\|}_{2}}^{2}\\ =2X\left(X^{T}W-Y^{T}\right)=2X\cdot X^{T}\cdot W-2X\cdot Y^{T} \end{array} \end{array}$$

Next, the derivative of $r_{1}{\sum_{k=1}^{k}\left\|W^{k}\right\|}_{F}$ with respect to *W*, when *k* = 1, assuming that this group has *m*_*k*_ total of SNPs, then:

$$\begin{array}{l} {\left\|W^{{}^{\prime }}\right\|}_{F}=\sqrt{{W_{idx_{1}-1}}^{2}+\cdots \text{+}{W_{idx_{1}-n}}^{2}+\cdots +{W_{idx_{s}-1}}^{2}+{W_{idx_{s}-2}}^{2}+{W_{idx_{s}-n}}^{2}} \end{array}$$

Where *idx*_1_⋯*idx*_*s*_ represents the index number of the SNP in the group,

$W=\left[\begin{array}{l} w_{11}w_{12}\cdots w_{1n}\\ w_{21}w_{22}\cdots w_{2n}\\ \cdots \cdots \cdots \cdots \cdots \\ w_{1}w_{m2}\cdots w_{mn} \end{array}\right]$ representsthe weight coefficient of m SNPs on *Y*_1_*Y*_2_⋯*Y*_*n*_.

$$\begin{array}{l} \begin{array}{c} \nabla_{W}\left(\overset{k}{\underset{k=1}{\sum }}{\left\|W^{k}\right\|}_{F}\right)=\\ \left[\begin{array}{c} \nabla_{w_{11}}\overset{k}{\underset{k=1}{\sum }}{\left\|W^{k}\right\|}_{F}\nabla_{w_{12}}\overset{k}{\underset{k=1}{\sum }}{\left\|W^{k}\right\|}_{F}\cdots \nabla_{w_{1n}}\overset{k}{\underset{k=1}{\sum }}{\left\|W^{k}\right\|}_{F}\\ \nabla_{w_{21}}\overset{k}{\underset{k=1}{\sum }}{\left\|W^{k}\right\|}_{F}\nabla_{w_{22}}\overset{k}{\underset{k=1}{\sum }}{\left\|W^{k}\right\|}_{F}\cdots \nabla_{w_{2n}}\overset{k}{\underset{k=1}{\sum }}{\left\|W^{k}\right\|}_{F}\\ \cdots \cdots \cdots \cdots \cdots \cdots \cdots \cdots \cdots \cdots \cdots \cdots \cdots \cdots \cdots \cdots \cdots \cdots \cdots \cdots \cdots \cdots \cdots \cdots \cdots \cdots \\ \nabla_{w_{m1}}\overset{k}{\underset{k=1}{\sum }}{\left\|W^{k}\right\|}_{F}\nabla_{w_{m2}}\overset{k}{\underset{k=1}{\sum }}{\left\|W^{k}\right\|}_{F}\cdots \nabla_{w_{mn}}\overset{k}{\underset{k=1}{\sum }}{\left\|W^{k}\right\|}_{F} \end{array}\right] \end{array} \end{array}$$

For $\nabla_{w_{11}}\overset{k}{\underset{k=1}{\sum }}{\left\|W^{k}\right\|}_{F}$ , suppose that the first SNP is in group *S*, then in the previous formula, only ${\left\|W^{k_{s}}\right\|}_{F}$ contains the weight coefficient of the SNP and *Y*.

Take the partial derivative with respect to *w*_11_:

$$\begin{array}{l} \nabla_{w_{11}}\overset{k}{\underset{k=1}{\sum }}{\left\|W^{k}\right\|}_{F}=\nabla_{w_{11}}{\left\|W^{s}\right\|}_{F}=\frac{1}{2{\left\|W^{s}\right\|}_{F}}▪2w_{11} \end{array}$$

The partial derivatives of the other terms can be derived and so on. We can find the derivative result:

$$\begin{array}{l} \begin{array}{c} \nabla_{W}\left(\overset{k}{\underset{k=1}{\sum }}{\left\|W^{k}\right\|}_{F}\right)\\ =\left[\begin{array}{c} \frac{1}{2{\left\|W^{s_{1}}\right\|}_{F}}\cdot 2w_{11}\frac{1}{2{\left\|W^{s_{1}}\right\|}_{F}}\cdot 2w_{12}\cdots \frac{1}{2{\left\|W^{s_{1}}\right\|}_{F}}\cdot 2w_{1n}\\ \frac{1}{2{\left\|W^{s_{2}}\right\|}_{F}}\cdot 2w_{21}\frac{1}{2{\left\|W^{s_{2}}\right\|}_{F}}\cdot 2w_{22}\cdots \frac{1}{2{\left\|W^{s_{2}}\right\|}_{F}}\cdot 2w_{2n}\\ \cdots \cdots \cdots \cdots \cdots \cdots \cdots \cdots \cdots \cdots \cdots \cdots \cdots \cdots \cdots \cdots \cdots \cdots \cdots \cdots \cdots \\ \frac{1}{2{\left\|W^{s_{k}}\right\|}_{F}}\cdot 2w_{m1}\frac{1}{2{\left\|W^{s_{k}}\right\|}_{F}}\cdot 2w_{m2}\cdots \frac{1}{2{\left\|W^{s_{k}}\right\|}_{F}}\cdot 2w_{mn} \end{array}\right] \end{array} \end{array}$$

Where *s*_1_⋯*s*_*k*_ represents that the SNP belongs to group *s*_*k*_, and ${\left\|W^{k_{s}}\right\|}_{F}$ represents the regular term of the group in which the SNP belongs.

$$\begin{array}{l} \nabla_{W}(\sum_{k=1}^{k}{\left\|W^{k}\right\|}_{F}\\ =\left[\begin{array}{llll} \frac{1}{2{\left\|W^{s_{1}}\right\|}_{F}} & & & \\ & \frac{1}{2{\left\|W^{s_{2}}\right\|}_{F}} & & \\ & & \ddots & \\ & & & \frac{1}{2{\left\|W^{s_{k}}\right\|}_{F}} \end{array}\right]\cdot \left[\begin{array}{l} w_{11}w_{12}\cdots w_{1n}\\ w_{21}w_{22}\cdots w_{2n}\\ \cdots \cdots \cdots \cdots \cdots \cdots \cdots \\ w_{m1}w_{m2}\cdots w_{mn} \end{array}\right]\cdot 2\\ =D\;\cdot \;W\;\cdot \;2 \end{array}$$

Let's take the derivative of $r_{2}{\sum_{i=1}^{d}\left\|W^{i}\right\|}_{2}$ with respect to *W* : where

$$\begin{array}{l} \;{\left\|w^{i}\right\|}_{2}=\sqrt{{w_{i1}}^{2}+{w_{i2}}^{2}+\cdots +{w_{in}}^{2}}\\ \begin{array}{c} \nabla_{W}\overset{d}{\underset{i=1}{\sum }}{\left\|w^{i}\right\|}_{2}\\ =\left[\begin{array}{c} \nabla_{w_{11}}\|w^{1}{\|}_{2}\nabla_{w_{12}}\|w^{1}{\|}_{2}\cdots \nabla_{w_{1n}}\|w^{1}{\|}_{2}\\ \nabla_{w_{21}}\|w^{2}{\|}_{2}\nabla_{w_{22}}\|w^{2}{\|}_{2}\cdots \nabla_{w_{2n}}\|w^{2}{\|}_{2}\\ \cdots \cdots \cdots \cdots \cdots \cdots \cdots \cdots \cdots \cdots \cdots \cdots \cdots \cdots \cdots \cdots \cdots \cdots \cdots \cdots \cdots \cdots \\ \nabla_{w_{m1}}\|w^{m}{\|}_{2}\nabla_{w_{m2}}\|w^{m}{\|}_{2}\cdots \nabla_{w_{mn}}\|w^{m}{\|}_{2} \end{array}\right] \end{array} \end{array}$$

For one of these terms, $\nabla_{w_{11}}{\left\|W^{1}\right\|}_{2}=\frac{1}{2{\left\|W^{1}\right\|}_{2}}▪2w_{11}$, the other terms can be analogous.

(16) $$\begin{array}{r} \begin{array}{c} \nabla_{W}\left(\overset{d}{\underset{i=1}{\sum }}{\left\|W^{i}\right\|}_{2}\right)\\ =\left[\begin{array}{c} \frac{1}{2{\left\|W^{1}\right\|}_{2}}\cdot 2w_{11}\frac{1}{2{\left\|W^{1}\right\|}_{2}}\cdot 2w_{12}\cdots \frac{1}{2{\left\|W^{1}\right\|}_{2}}\cdot 2w_{1n}\\ \cdots \cdots \cdots \cdots \cdots \cdots \cdots \cdots \cdots \cdots \cdots \cdots \cdots \cdots \cdots \cdots \cdots \cdots \cdots \cdots \cdots \cdots \\ \frac{1}{2{\left\|W^{m}\right\|}_{2}}\cdot 2w_{m1}\frac{1}{2{\left\|W^{m}\right\|}_{2}}\cdot 2w_{m2}\cdots \frac{1}{2{\left\|W^{m}\right\|}_{2}}\cdot 2w_{mn} \end{array}\right] \end{array} \end{array}$$

Equation (16) can be written as:

$$\begin{array}{l} \nabla_{W}(\sum_{i=1}^{d}{\left\|W^{i}\right\|}_{2}\\ =\left[\begin{array}{llll} \frac{1}{2{\left\|W^{1}\right\|}_{2}} & & & \\ & \frac{1}{2{\left\|W^{2}\right\|}_{2}} & & \\ & & \ddots & \\ & & & \frac{1}{2{\left\|W^{m}\right\|}_{2}} \end{array}\right]\cdot \left[\begin{array}{l} w_{11}w_{12}\cdots w_{1n}\\ w_{21}w_{22}\cdots w_{2n}\\ \cdots \cdots \cdots \cdots \cdots \\ w_{m1}w_{m2}\cdots w_{mn} \end{array}\right]\cdot 2\\ =\tilde{D}\;\cdot \;W\;\cdot \;2 \end{array}$$

Then, equation (14) can be obtained as equation (17).

(17) $$\begin{array}{r} W={\left(XX^{T}+\gamma_{1}D+\gamma_{2}\tilde{D}\right)}^{-1}XY^{T} \end{array}$$

Therefore, *W* can be obtained effectively by solving linear equation $\left(XX^{T}+\gamma_{1}D+\gamma_{2}\tilde{D}\right)W=XY^{T}$, which improves the efficiency of solving group sparse model.

In this study, the above group sparse model is used as the feature selection in the three-stage framework. After the implementation of this stage, each SNP has a weight vector corresponding to each ROI, and the importance of SNP can be judged according to the weight in the feature vector, and then the feature SNP can be selected.

### Support Vector Regression

In this paper, the three-stage analysis framework uses the support vector regression model to predict the phenotype value of ROI. Here, the support vector regression model is briefly introduced. Support vector machine (SVM) can be used to solve problems such as classification and regression. The response variable of classification problem is discrete tag value, while the response variable value of regression problem is usually continuous. Support vector regression (SVR) is an extension of SVM on regression tasks (Huang et al., 2018). Support vector regression can be roughly divided into linear support vector regression and non-linear support vector regression.

For the linear support vector regression problem, given the training set:

$$\begin{array}{l} D=\left\{\left(x_{1},x_{2}\right),\cdots \left(x_{i},y_{i}\right)\right\},x\in R^{n},y\in R \end{array}$$

construct a linear function *f* (*x*) = 〈*w* · *x*〉 + *b* to fit *D*, as far as possible to make the precision ε as flat as possible, which is equivalent to minimizing $\frac{1}{2}{\left\|w\right\|}^{2}$, on the other hand, the reconstruction error is required to be as small as possible. Therefore, it is transformed into the problem of solving constraint optimization. The fitting function can be obtained by using Lagrange theorem quadratic programming and duality principle:

(18) $$\begin{array}{r} f\left(x\right)=\overset{l}{\underset{i=1}{\sum }}\left(\alpha_{i}^{*}-a_{i}\right)\left(x_{i}\cdot x\right)+b \end{array}$$

For the non-linear regression problem, the solution is to transform the non-linear problem in the low dimensional space into the linear regression problem in the high dimensional space. The solution of the non-linear regression function (19) is expressed by the support vector, so the support vector can be regarded as the expansion of the kernel function.

(19) $$\begin{array}{r} f\left(x\right)=\overset{l}{\underset{i=1}{\sum }}\left(\alpha_{i}^{*}-\alpha_{i}\right)K\left(x_{i}\cdot x\right)+b \end{array}$$

The kernel function is needed to map the non-linear function in low dimensional space to the linear function in high dimensional space. The kernel function is to compute the vector first in a low dimensional space and then compute the inner product of the vector in a higher dimensional space. It can be seen that the selection of appropriate kernel function is the key element of SVR, and common kernel functions include polynomial kernel, neural network kernel and gaussian radial basis function kernel. The regression prediction stage of the three-stage analysis framework in this paper adopts the SVR model, which receives the characteristic SNPs output in the feature selection stage, and then trains the SVR model on the data set, so as to realize the regression prediction of SNP and ROI.

## Experimental Results

### Clustering Results

Clustering algorithm parameters usually affect clustering results, such as clustering number, distance formula, etc. In this paper, different clustering parameters are compared and analyzed, and then appropriate parameters are selected to obtain more stable clustering results. Under different hierarchical clustering methods, the composite correlation coefficient between hierarchical clustering tree and distance vector is calculated as shown in Table 1.

**Table 1:** Cluster distance parameter setting.

	**The shortest distance method**	**Maximum distance method**	**Centroid distance method**	**Unweighted mean distance**	**Least variance method**
Euclidean distance	0.7134	0.5346	0.5938	0.5196	0.4917
Absolute distance	0.7096	0.5255	0.4448	0.6124	0.4921
Minkowski distance	0.8545	0.7685	0.7973	0.8025	0.7288
Variance weighted distance	0.7118	0.5410	0.5595	0.6479	0.5403

When the composite correlation coefficient is closer to 1, the clustering is more ideal. It can be seen that when the distance calculation method adopts the Minkowski distance (parameter r is tested and 0.23 is the best) and the shortest distance method is adopted for the hierarchical clustering method, the correlation coefficient can reach 0.8545, which is the closest to 1, that is, the clustering effect is the most ideal under this method.

### Comparison of Regression Analysis Results of Different Evaluation Indexes

**Figure 2:**
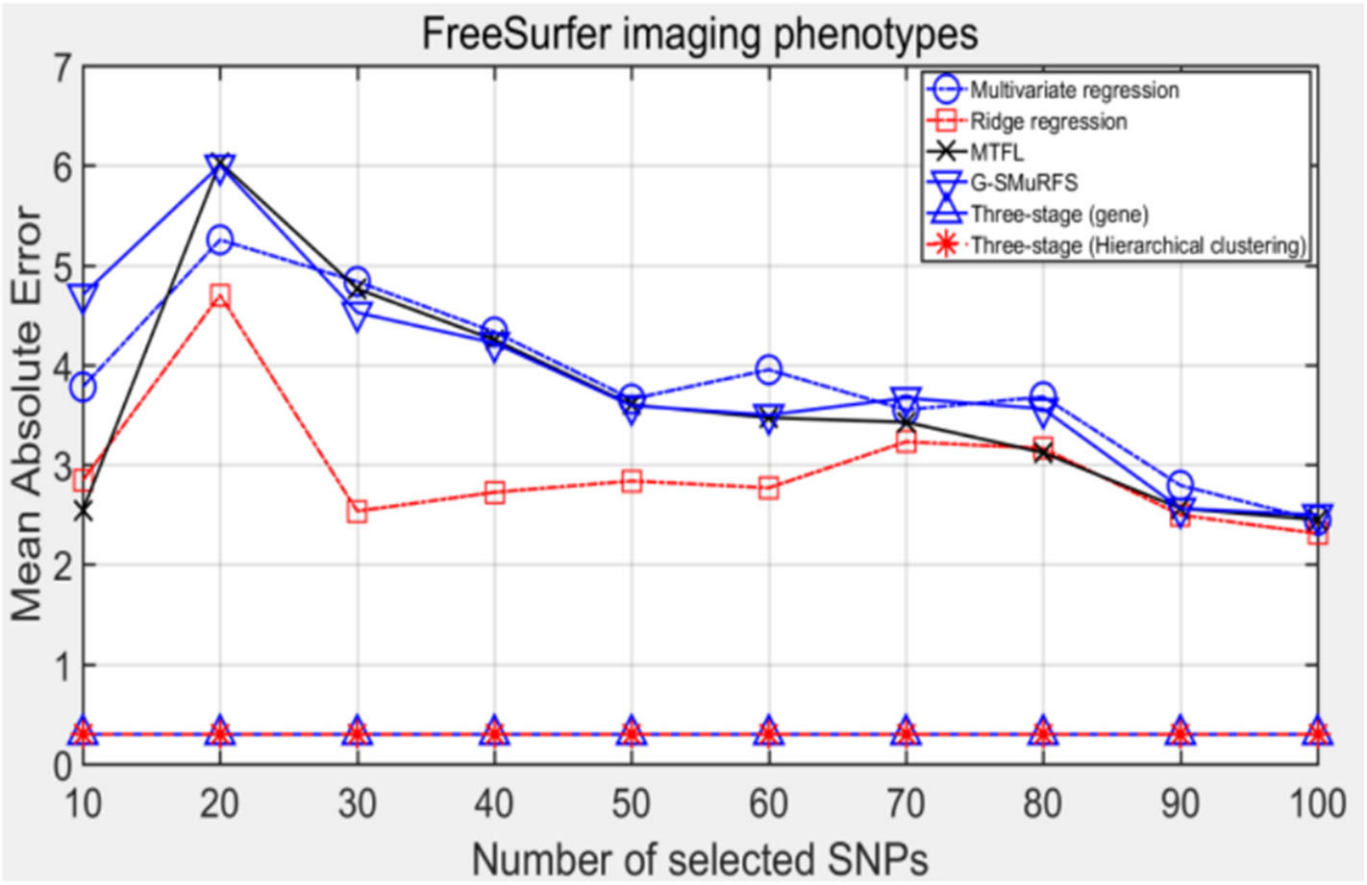
The comparison results on MAE.

**Figure 3:**
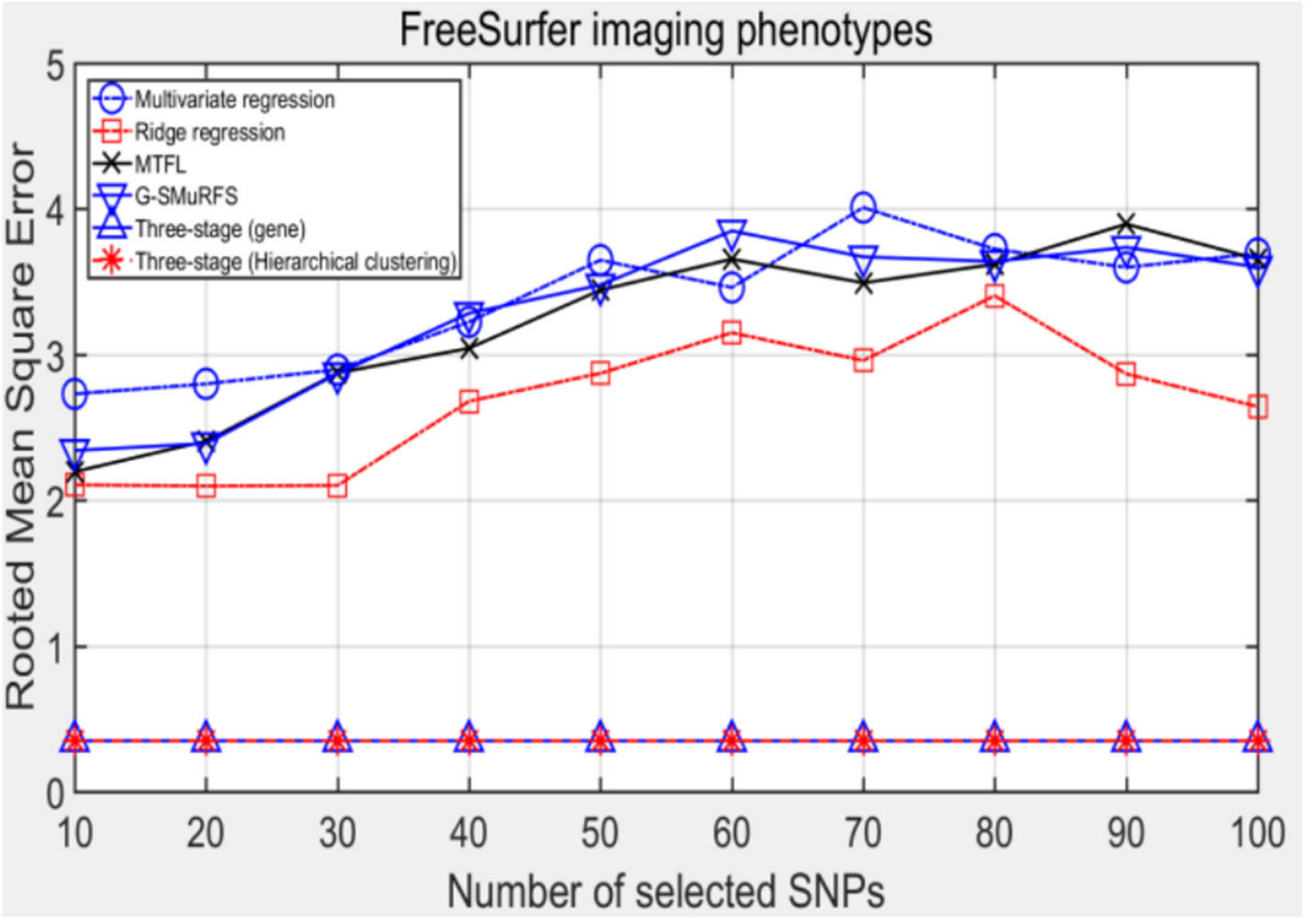
The comparison results on RMSE.

The following methods were compared: the three-stage analysis framework proposed in this paper and ridge regression, multivariate regression, sparse model MTFL and group sparse method G-SMuRFS. The results are shown in Figure 2, where 'three-stage (gene)' represents the prior information of gene in the sparse group model. The SNPs located in the same gene are divided into one group, while 'three-stage' means that different groups are classified by Hierarchical clustering. As we can see from Figure 3, the previous four are the methods proposed by previous researchers, and the last two are the three-stage analysis framework that we proposed, SNPs were grouped by genetic and hierarchical clustering separately. On FreeSurfer imaging phenotypes testing, the last two we proposed three-stage analysis framework outperforms the previous four approaches both in terms of prediction accuracy and robustness.

**Figure 4:**
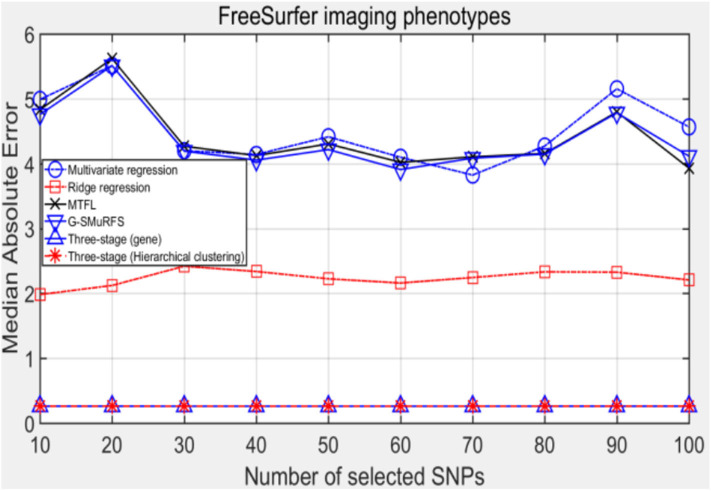
The comparison results on MeAE.

MeAE evaluation index can usually be used to eliminate the interference of outliers, and the results are shown in Figure 4.

*R*^2^ is the ratio of the sum of the squares of the regression to the total sum of the squares in the multivariate regression. It is a statistic to measure the degree of fitting and reflects the proportion explained by the regression equation estimated in the variation of the dependent variable *y*. The closer *R*^2^ is to 1, the greater the proportion of the regression sum of squares in the total sum of squares, and the closer the regression line is to the observation points, the better the regression fitting degree will be. The results are shown in Figure 6.

**Figure 5:**
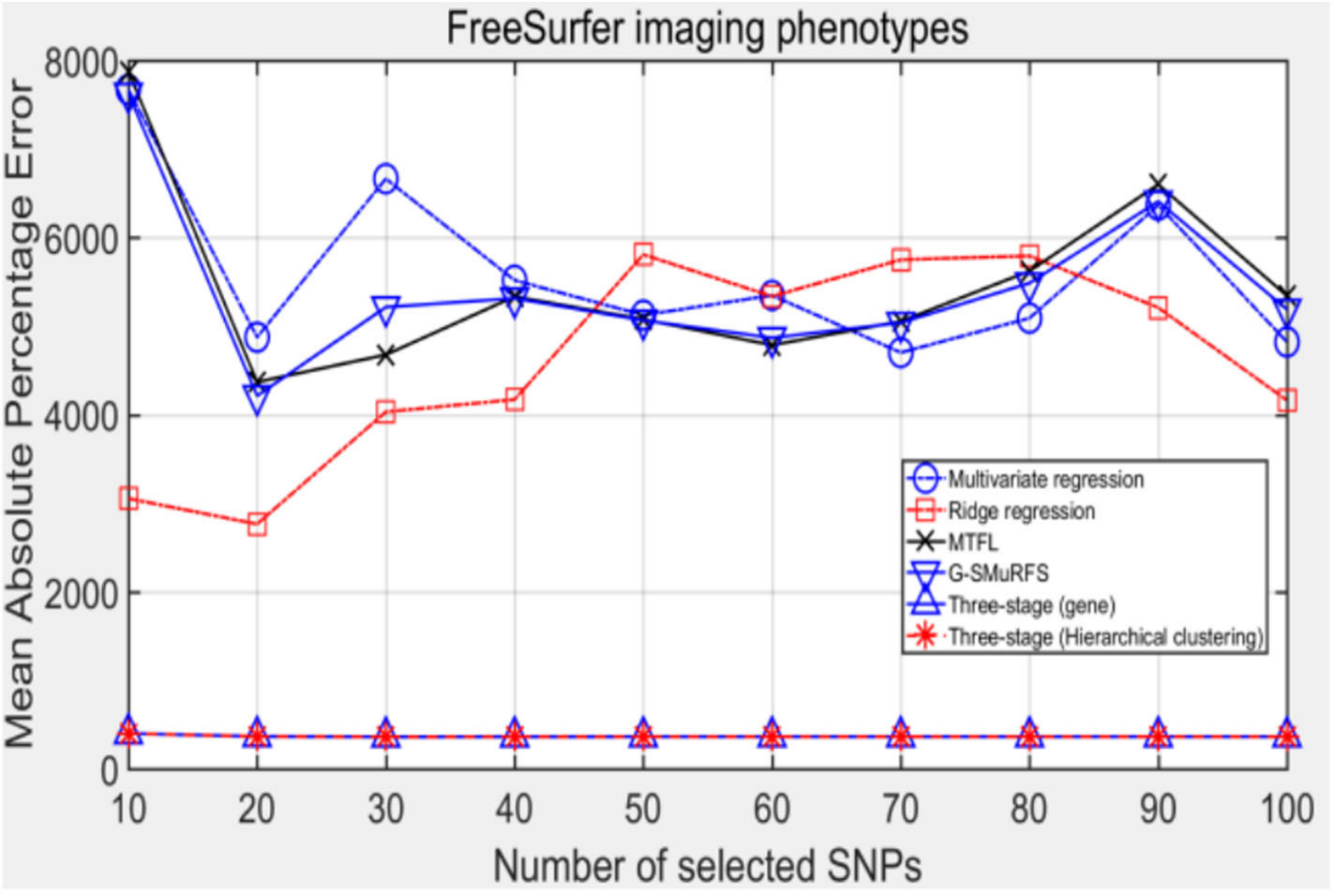
The comparison results on MAPE.

**Figure 6:**
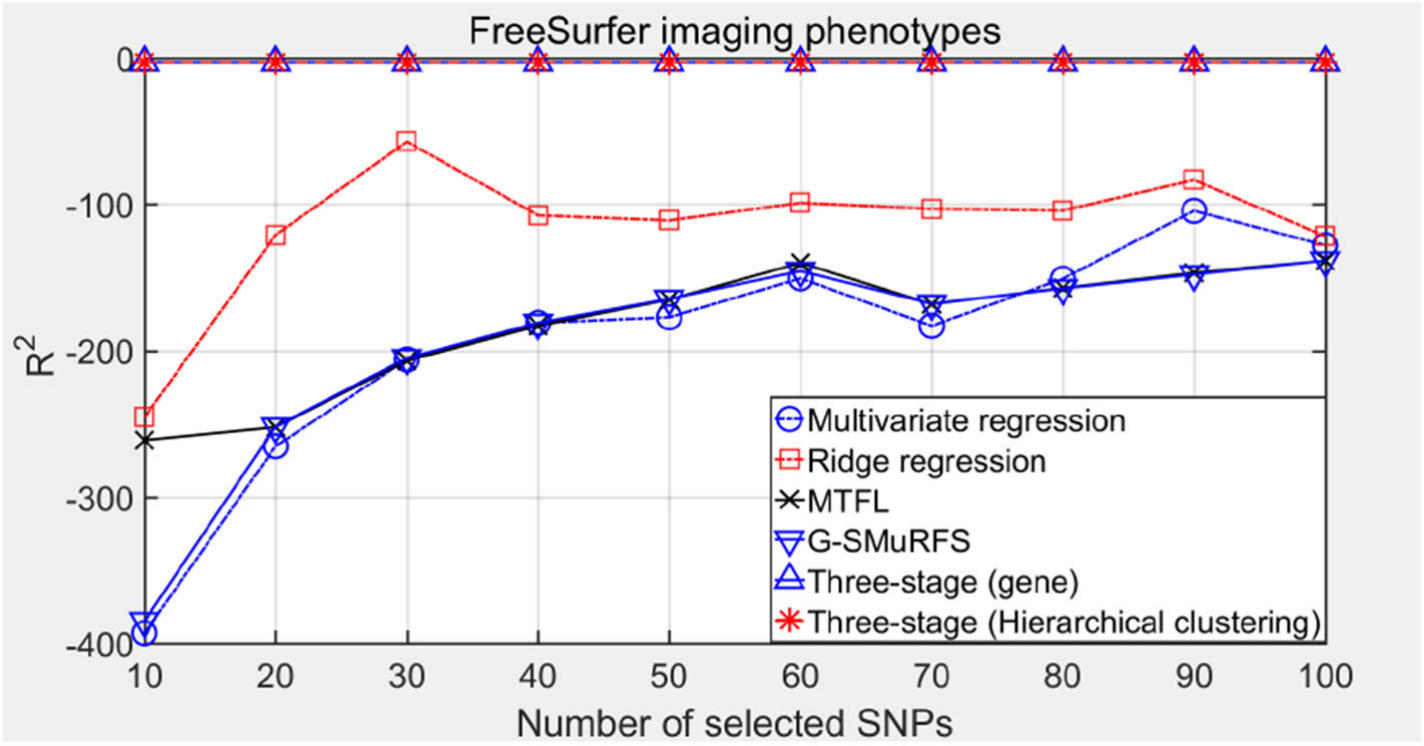
The comparison results on R^2^.

**Figure 7:**
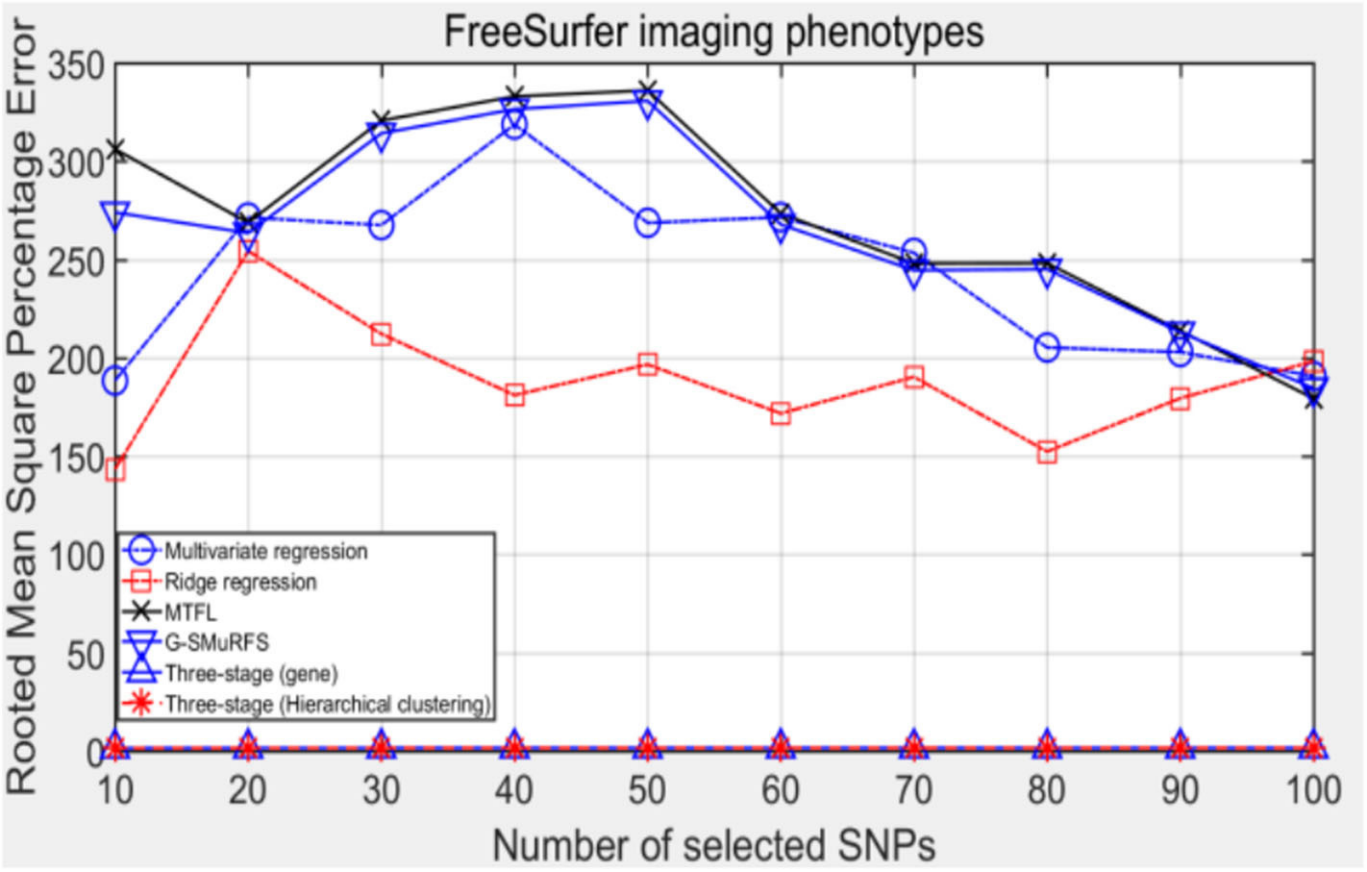
The comparison results on RMSPE.

Comparing the experimental results of Figures 2–7, it is found that the regression error of the three-stage analysis framework proposed in this paper is significantly smaller than that of other methods under different indexes. Note that the regression error of other methods changes greatly with the change of the number of SNPs, and the error becomes larger even when the number of SNPs increases. However, it can be found that the regression results of our method are relatively stable, which is reflected in that the error value does not change with the increase of the number of SNPs selected, which indirectly indicates that the group sparse model can steadily select the most representative SNP from all the candidate SNPS. In addition, SVR may also contribute the stability of our method.

For the multi-regression task, the same SNPs subset may have different significance for different ROIs, so the prediction accuracy of each ROI may be different. Next, the prediction performance is compared and analyzed from a single ROI.

### Comparative Analysis of Single ROI Value

**Figure 8:**
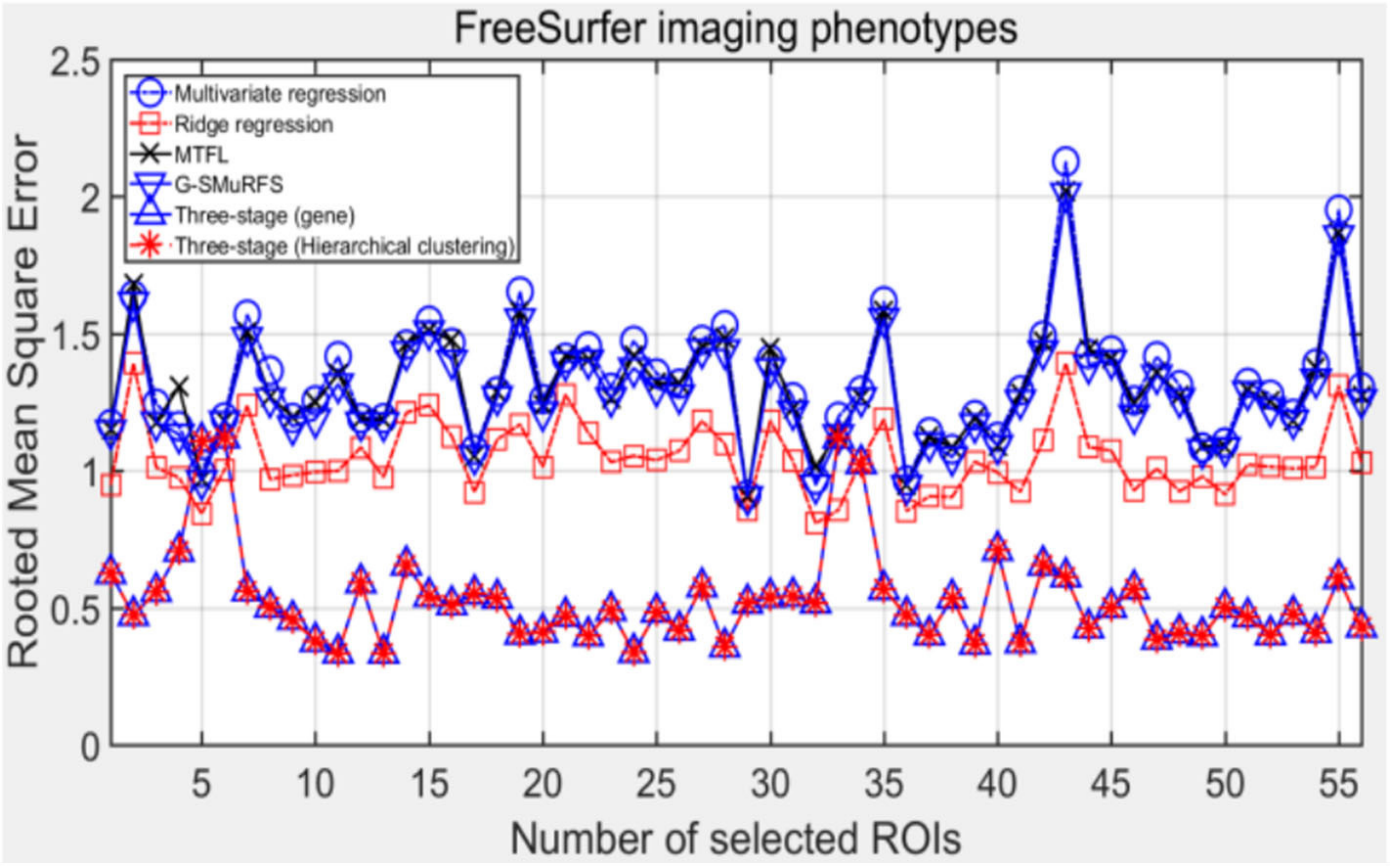
The comparison results on single ROI.

Brain region location, structure, size and metabolic level corresponding to different ROIs have different effects on the judgment of Alzheimer's disease, and different ROIs may be influenced by different functional genes. Therefore, the next step is to further demonstrate the regression performance of different methods on different ROIs, as shown in Figure 8.

The results in the figure show that some ROIs are highly correlated with selected SNP characteristics and can be predicted well-under most regression methods. However, some ROI regions are not highly correlated with the selected SNP characteristics, resulting in large differences in the accuracy of their predictions. The experimental results show that the proposed analytical framework is superior to other methods.

## Conclusion

So far, some risk genes that are significantly associated with AD have been excavated from the genomic level, but this may still be only the tip of the iceberg behind their complex genetic mechanisms. This paper proposes a framework of SNPs associated with ROI analysis of three phase, the framework uses clustering algorithm to remove two loci potential linkage disequilibrium structure, and then use the priori information such as gene group sparse model structure, linkage disequilibrium structure selected characteristics of SNP, finally use regression model to analyze the connection degree of characteristics between SNPs and ROI. From the perspective of multiple measures, our method has certain advantages in identifying ROI-related characteristic SNP sites, which means that mutations in these sites may lead to changes in relevant functional genes, thus affecting the function of brain regions.

The proposed three-stage analysis framework was compared with ridge regression, multivariate regression, sparse model MTFL and group sparse method G-SMuRFS under different indexes. It can be seen that the regression error of the three-stage analysis framework proposed in this paper is significantly smaller than that of other methods, and the results of this method are relatively stable, which indirectly indicates that the group sparse model can stably select the most representative SNP as the features from the candidate SNPs. For the multi-regression task, the importance of the same SNP subset to different ROIs is different. The correlation analysis between a single ROI and the selected SNP characteristics is conducted by different methods. The experimental results show that the analysis framework proposed in this paper has some advantages over other methods in general.

The occurrence and development of AD are related to the interaction of multiple biomolecules. If only one level of omics data is studied, it will deviate from the real disease model and ignore the real and complete risk factors, resulting in the lack of heritability. Therefore, the further work will be: (1) the multi-mode brain image data contains more information, and the data of different modes have complementary information. The establishment of multi-mode brain image data fusion analysis model is conducive to the accurate identification of early AD patients; (2) on the basis of in-depth mining of AD genome-wide SNP data, the integration of other levels of omics data is conducive to a systematic and complete understanding of the occurrence and development process of AD.

## Data Availability Statement

Data Publicly available datasets were analyzed in this study. This data can be found here: adni.loni.ucla.edu.

## Author Contributions

XL, JZ, YQ, and SC carried out the design of the study and performed the statistical analysis. JZ contributed to examination of algorithms and contributed to downstream analysis. YQ and LL implemented the experiments and analyzed the results. HL, HC, and SL participated in software coding and helped to draft the manuscript. All authors read and approved the final manuscript.

## Conflict of Interest

The authors declare that the research was conducted in the absence of any commercial or financial relationships that could be construed as a potential conflict of interest.
